# Long-term changes in glucose metabolism after gestational diabetes: a double cohort study

**DOI:** 10.1186/1471-2393-14-296

**Published:** 2014-08-30

**Authors:** Hanna Huopio, Heidi Hakkarainen, Mirja Pääkkönen, Teemu Kuulasmaa, Raimo Voutilainen, Seppo Heinonen, Henna Cederberg

**Affiliations:** Department of Pediatrics, Kuopio University Hospital, P.O.Box 100, FI-70029 KYS Kuopio, Finland; Department of Obstetrics and Gynecology, Kuopio University Hospital, Kuopio, Finland; Siilinjärvi Health Center, Siilinjärvi, Finland; Department of Medicine, University of Eastern Finland, Kuopio, Finland; Department of Medicine, University of Eastern Finland and Kuopio University Hospital, Kuopio, Finland

**Keywords:** Gestational diabetes, Type 2 diabetes, Insulin secretion, Insulin resistance, Obesity

## Abstract

**Background:**

Gestational diabetes (GDM) has been associated with an elevated risk of type 2 diabetes in women after the pregnancy. Recognition of the factors differentiating the women at highest risk of progression to overt disease from those who remain normoglycemic after gestational diabetes is of key importance for targeted prevention programmes. To this aim, we investigated the incidence and risk factors of prediabetes and type 2 diabetes with a view to the underlying pathophysiological mechanisms in a long-term follow-up of women with a history of gestational diabetes.

**Methods:**

489 women with GDM and 385 normoglycemic controls attended a follow-up study after pregnancy (mean follow-up time 7.3, SD 5.1 years) in Kuopio, Finland. Glucose tolerance was evaluated with an oral glucose tolerance test, insulin sensitivity by Matsuda insulin sensitivity index (ISI), and insulin secretion by Disposition Index 30 (DI30).

**Results:**

GDM increased risk of pre-diabetes and diabetes (HR 3.7, 95% C.I. 2.8-4.7 and HR 40.7, 95% C.I. 5.3-310.1, respectively, after adjustment for confounding factors) and was associated with both increased fasting (*P* < 0.001) and 2-hour plasma glucose (*P* < 0.001) during OGTT at the follow-up study. This effect was attenuated when adjusted for Matsuda ISI but abolished after adjustments with DI30 suggesting insulin secretion is the key defect leading to type 2 diabetes after GDM pregnancy. Increase in waist circumference and weight after pregnancy predicted the development of hyperglycemic conditions in women with a history of GDM (*P* < 0.001, and *P* = 0.002, respectively).

**Conclusions:**

Pre-diabetic stages after GDM pregnancy are frequent and reflect the progressive risk of type 2 diabetes in long-term follow-up. Hyperglycemia after GDM pregnancy results from beta cell failure and inability to compensate the increased insulin resistance by insulin secretion. Importantly, increase in waist circumference and as well as weight gain during the follow-up is associated with progression to prediabetes and type 2 diabetes in women with a history GDM.

## Background

Gestational diabetes (GDM) is defined as glucose intolerance detected for the first time during pregnancy. The prevalence of GDM is on the increase and currently affects 2–14% of all pregnancies [1, 2], and is associated with markedly elevated risk of both maternal and fetal complications [3]. During pregnancy, insulin resistance increases and when accompanied by impaired beta cell function, the risk of GDM increases. After the pregnancy glucose tolerance normalizes, but previous studies have demonstrated that affected women have at least a seven-fold risk of developing type 2 diabetes later in life [4–7].

Impaired insulin secretion and insulin resistance are the two main metabolic disturbances in the pathogenesis of type 2 diabetes and often coexist [6]. Previous studies have shown that type 2 diabetes usually develops due to insufficient pancreatic insulin secretion to compensate for the existing insulin resistance. Insulin resistance is present early in the natural history of type 2 diabetes and marked beta-cell dysfunction is a rather late event [8]. Both insulin resistance and beta-cell dysfunction are also features of GDM. Both of these disturbances in glucose metabolism are influenced by genetic and environmental factors [9], but behavioural and environmental factors like obesity and physical inactivity are emphasized in the development of insulin resistance while genetic factors have a stronger influence on the insulin secretion [10].

Type 2 diabetes is preceded by a pre-diabetic stage characterized by mild-to-moderate elevation of fasting and/or postprandial glucose levels [11]. Impairment in insulin sensitivity is present already at relatively low plasma glucose levels within the normoglycemic range, years before the development of overt type 2 diabetes. Pre-diabetic states, defined by an oral glucose tolerance test (OGTT), include isolated impaired fasting glucose (IIFG), isolated impaired glucose tolerance (IIGT), or a combination of these (IFG + IGT) [6], and represent metabolic states in the continuum between normal glucose tolerance and diabetic hyperglycemia. Both IFG and IGT similarly predict incident diabetes, with the highest risk in individuals with combined IFG and IGT [11]. Previous studies have shown that up to 60% of individuals who have either IGT or IFG will subsequently develop overt diabetes. IFG and IGT differ metabolically and therefore identify different risk groups for impaired glucose regulation. The inability to maintain adequate basal insulin secretion and to control hepatic glucose output is characteristic for IIFG. In contrast, IIGT is associated with peripheral insulin resistance, especially at the level of skeletal muscle [11].

Compelling evidence from randomized controlled lifestyle intervention trials has shown that up to 58% reduction in the risk of type 2 diabetes can be achieved by dietary modifications and increased physical activity in individuals with IGT [12, 13]. Therefore, well-organized follow-up of women with a history of GDM and timely diagnosis of pre-diabetic states are crucial for targeted intervention to prevent development of overt type 2 diabetes. In this current study, we evaluated glucose tolerance in women after GDM pregnancy (N = 489) and in healthy controls (N = 385) in a longitudinal long-term follow-up study, and investigated the pathophysiological mechanism and risk factors underlying the deterioration of glucose tolerance and development of prediabetes and type 2 diabetes following GDM pregnancy.

## Methods

The study population was collected from an existing clinical pregnancy registry at the Kuopio University Hospital, Kuopio, Finland. All patients who had OGTT during pregnancy between 1989 and 2009 were contacted by a letter and invited for the study. A total of 489 previously non-diabetic women were identified who were diagnosed with GDM between the years 1989 and 2009; 385 women with normal glucose tolerance in an oral glucose tolerance test (OGTT, 75 g glucose dose after overnight fasting) during pregnancy served as controls. Women with a diagnosis of type 1 diabetes or late-onset autoimmune diabetes (LADA) were excluded from the study. For patients with more than one delivery during the study period, the first pregnancy with GDM was considered as the index pregnancy. Women with multiple pregnancy were excluded. Among participants with GDM, 71.6% had GDM in one pregnancy, 21.2% in two pregnancies, 6.3% in three pregnancies and 0.9% in four pregnancies. The diagnosis of GDM was based on the contemporary criteria: fasting blood glucose > 4.8 mmol/l, 1- hour blood glucose >10.0 mmol/l and 2-hour blood glucose >8.7 mmol/l until September 2001, and since September 2001 fasting plasma glucose (FPG) > 4.8 mmol/l, 1-hour plasma glucose (PG) >11.2 mmol/l and 2-hour plasma glucose >9.9 mmol/l. One or more elevated values during an OGTT resulted in the diagnosis of GDM.

### Follow-up study

All participants attended a 1-day visit at the Kuopio University hospital between years 2006 and 2009. Data on background characteristics and lifestyle was collected with questionnaires and interview with a trained study nurse. Smoking status was defined as current smoking (yes vs. no). Physical activity (physically active vs. inactive) refers to leisure time exercise (physically active, regular exercise at least 30 min 1 or 2 times per week vs. physically inactive, occasional exercise or no exercise). Data on all pregnancies was obtained with a questionnaire and parity was defined as the total number of pregnancies.

#### Clinical measurements

Height and weight were measured to the nearest 0.5 cm and 0.1 kg, respectively. BMI was calculated as weight (kg) divided by height (m) squared.

#### OGTT

To study the glucose tolerance after the pregnancy, a 2-hour OGTT (75 g of glucose) was performed in all women (mean follow-up 7.3, SD 5.1 years), and samples for plasma glucose and insulin were drawn at 0, 30, and 120 min. Glucose tolerance was evaluated based on OGTT as follows: NGT (FPG < 5.6 mmol/l and 2-h PG < 7.8 mmol/l), isolated IFG (IIFG) (FPG 5.6-6.9 mmol/l and 2-h PG < 7.8 mmol/l), isolated IGT (IIGT)(FPG < 5.6 mmol/l and 2-h PG 7.8-11.0 mmol/l), IFG and IGT(IFG + IGT) FPG 5.6-6.9 mmol/l and 2-h PG 7.8-11.0 mmol/l), and newly diagnosed type 2 diabetes (FPG ≥ 7.0 mmol/l and/or 2-h PG ≥ 11.1 mmol/l)[6]. Prediabetes encompasses both isolated IFG and isolated IGT as well as their combination. Fifteen women had started an anti-diabetic medication between the index pregnancy and the follow-up study visit and 14 were diagnosed with incident type 2 diabetes at the follow-up study visit.

#### Laboratory determinations

Plasma glucose was measured by enzymatic hexokinase photometric assay (Konelab Systems reagents; Thermo Fischer Scientific, Vantaa, Finland). Insulin was determined by immunoassay (ADVIA Centaur Insulin IRI no. 02230141; Siemens Medical Solutions Diagnostics, Tarrytown, NY). HbA1c was measured using the high-performance liquid chromatography assay (TOSOH G7 glycohemoglobin analyzer, Tosoh Bioscience Inc, San Francisco, CA), calibrated to DCCT standard. The trapezoidal method was used to calculate glucose area under the curve (AUC) and insulin AUC during the OGTT. Calculation of insulin sensitivity (Matsuda ISI), insulin secretion (InsAUC0-30/GluAUC0-30) and disposition indices (DI30) have been previously described [14].

### Statistical analysis

All statistical analyses were performed using SPSS 19 statistical software (SPSS, Chicago, IL). Anthropometric and biochemical variables were log-transformed to correct for their skewed distribution as appropriate. Comparisons between women with GDM and corresponding controls, as well as comparisons between the GDM subjects with abnormal glucose tolerance and NGT (mean, standard deviation, SD) were done using t-test for continuous and Chi^2^ test for categorical variables. The risk of prediabetic stages (including IIFG/IIGT/IFG + IGT) in GDM patients and controls was evaluated by Cox proportional hazards regression models, and adjusted for age, BMI, parity, the traditional risk factors for type 2 diabetes including current smoking and physical activity (physically active, regular exercise at least 30 min a week vs. physically less active, occasional exercise or no exercise). The risk of incident type 2 diabetes in patients with GDM and controls was compared with Cox proportional hazards regression model. Linear regression analysis was used to evaluate the association of GDM status in index pregnancy with hyperglycemia at follow-up, and adjusted for age and BMI at follow-up study, follow-up time, parity, current smoking, and physical activity. The participants with type 2 diabetes diagnosed during the follow-up time and started on anti-diabetic medication (N = 15) were not included in the linear regression analysis. *P* < 0.05 was considered statistically significant.

### Ethical considerations

The study was approved by the local ethics committee of the Kuopio University Hospital, and it was conducted in accordance with the Helsinki Declaration. All study participants gave written informed consent.

The study has adhered to the STROBE guidelines for observational studies.

## Results

The clinical and laboratory characteristics of the study groups are presented in Table 1. Participants with GDM were older (*P* < 0.001) and had higher BMI than the controls in the first trimester of the index pregnancy (*P* < 0.001). 36.6% of the GDM group had their first pregnancy as an index pregnancy whereas the corresponding rate was 54.5% in the control group (P < 0.001). The mean total number of pregnancies were 1.1 (SD = 1.3) and 0.7 (SD = 0.9) in women with a history of GDM and control women, respectively. No differences were observed in the duration of the pregnancy or in the birth weight of the child between the study groups. The follow-up time was shorter in the GDM patients as compared to controls (*P* < 0.001). At the follow-up study visit, GDM patients had higher BMI (*P* < 0.001), HbA1c (*P* < 0.001), glucose and insulin levels during an OGTT, and they were more insulin resistant than the control subjects (Matsuda ISI, *P* < 0.001). Insulin sensitivity-corrected insulin secretion (DI30) was significantly decreased in the GDM subjects (*P* < 0.001) as compared to the controls.

**Table 1:** **Clinical characteristics of the women with gestational diabetes and controls at index pregnancy and the follow-up study**

	GDM patients (n = 489)		Controls (n = 385)		
	Mean	SD	Mean	SD	***P***
***Index pregnancy***					
Age at delivery (yrs)	32.0	5.9	29.6	5.3	**<0.001**
Parity	1.1	1.3	0.7	0.9	**<0.001**
Primipara/multipara (%)	36.6/63.4		54.5/45.6		**<0.001**
Waist (cm)	91.3	13.4	86.3	11.7	**<0.001**
BMI in the first trimester (kg/m2)	26.4	4.8	24.1	3.8	**<0.001**
Duration of the pregnancy (weeks)	39.3	1.5	39.5	1.7	0.095
Birth weight of the child (g)	3648	545	3579	570	0.065
Follow-up study*					
Age at follow up (yrs)	37.8	7.2	38.4	6.3	0.200
Follow-up time (yrs)	6.0	4.4	9.0	5.5	**<0.001**
Family history of diabetes (%)	81.0		71.5		**0.001**
Current smoker (%)	18.8		13.6		**0.040**
BMI (kg/m^2^)	28.4	5.5	26.9	4.9	**<0.001**
HbA1c [mmol/mol (%)]	36 (5.5)	5	35 (5.3)	3	**<0.001**
Fasting plasma glucose (mmol/l)	5.6	0.5	5.3	0.4	**<0.001**
30-min plasma glucose (mmol/l)	8.0	1.7	7.1	1.5	**<0.001**
2-h plasma glucose (mmol/l)	6.0	1.7	5.5	1.2	**<0.001**
Fasting plasma insulin (pmol/l)	79.2	55.6	63.2	43.1	**<0.001**
30-min plasma insulin (pmol/l)	507.0	355.6	439.0	266	**0.013**
2-hour plasma insulin (pmol/l)	351.4	327.1	278.5	242.4	**<0.001**
P-glucose area under the curve (mmol/l*min)	832.6	160.3	749.6	130.6	**<0.001**
P-insulin area under the curve (pmol/l*min)	40880.3	28499.6	34414	21656	**<0.001**
HOMA-IR	2.9	2.1	2.2	1.6	**<0.001**
Matsuda ISI (mg/dl, mU/l)	5.7	3.2	7.1	3.6	**<0.001**
Disposition Index	167.4	65.1	206.3	77.6	**<0.001**

*Individuals diagnosed with type 2 diabetes during the follow-up time (n = 15) were not included in HbA1C and oral glucose tolerance tests. HOMA-IR, homeostatic model assessment of insulin resistance. P-values < 0.05 are indicated in bold font.

**Figure 1:**
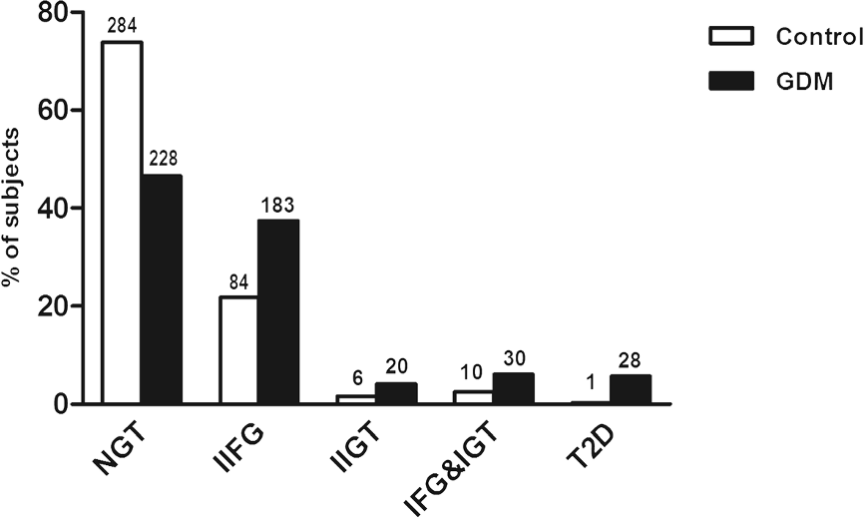
**Glucose tolerance of the control (white columns) and GDM subjects (black columns) evaluated during the follow-up visit (NGT = normal glucose tolerance, IIFG = isolated increased fasting glucose, IIGT = isolated impaired glucose tolerance, IFG & IGT = combination of IFG and IGT, type 2 diabetes).** The total numbers of study persons in each group are shown in numeric form above each column.

**Figure 2:**
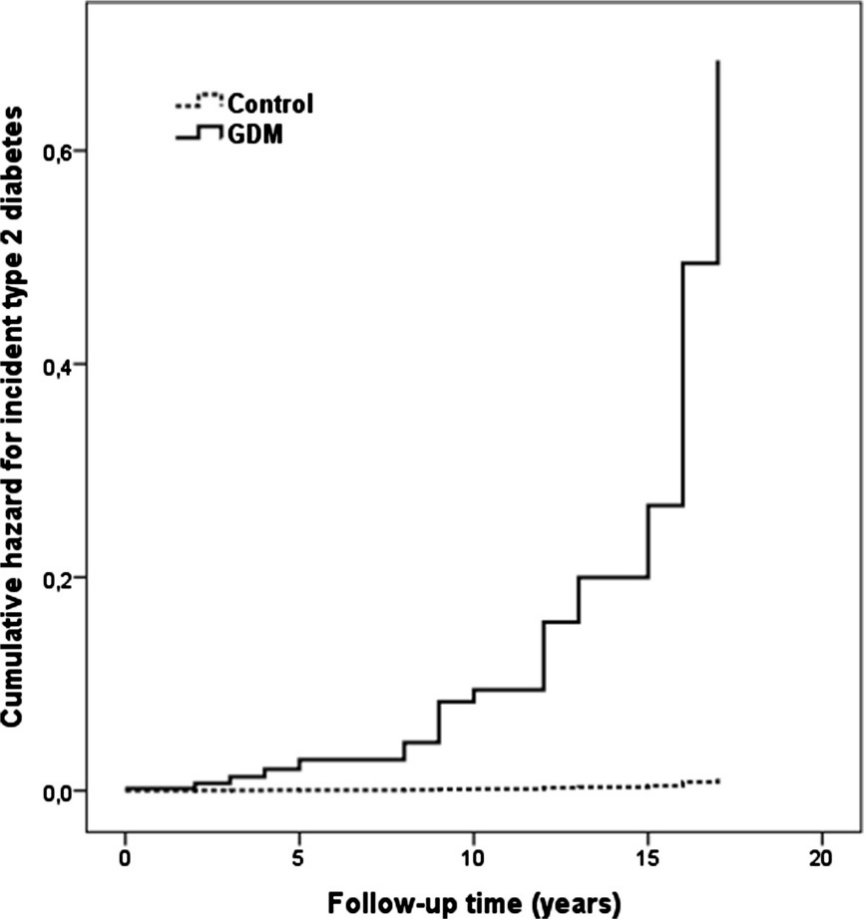
**The cumulative hazard for incident type 2 diabetes (the time point of diagnosis) during the course of the follow-up time in the controls (broken line) and GDM subjects (black line).**

The distribution of the GDM and control subjects according to the state of glucose tolerance in the OGTT at the follow-up study visit is shown in Figure 1. 47% of the GDM subjects and 74% of the controls had normal glucose tolerance at the follow-up study visit (*P* < 0.001). Prediabetes (isolated IFG, isolated IGT, or IFG& IGT) was detected in 48% of the GDM subjects and 26% of the controls (*P* 0.001). Isolated impaired fasting glucose (IIFG) was found in 37 vs. 22% (*P* < 0.001), isolated impaired glucose tolerance (IIGT) in 4 vs. 2% (*P* = 0.001), and IFG + IGT in 6 vs. 3% (*P* < 0.001) of GDM subjects and controls, respectively. During the follow-up time after the index pregnancy, none of the normoglycemic controls had proceeded to diabetes, whereas 15 (3.1%) subjects in the GDM group had been diagnosed with type 2 diabetes and started on anti-diabetic medication. At the follow-up visit, diabetes was diagnosed by OGTT in one (0.3%) control and further 13 (2.7%) GDM subjects. Figure 2 illustrates the risk of developing diabetes (the time point of diagnosis) during the course of the follow-up time (HR 40.7, 95% CI 5.3-310.1). The risk was minimal and linear in the control population but increased markedly in the GDM group especially after 10 years of follow-up.

The association between GDM and glucose tolerance at the follow-up study was evaluated by Cox proportional hazard model (Table 2). GDM increased the risk of pre-diabetes [(HR 4.0 (95% C.I. 3.1–5.1)] in all categories of glucose tolerance status [(IIFG: HR 3.1 (95% C.I. 2.4-4.0), IIGT: HR 7.8 (95% C.I. 2.9-21.6), IFG + IGT: HR 4.7 (95% C.I. 2.2-10.0)]. The pre-diabetes-increasing risk of GDM remained significant after the adjustment for age, BMI, parity, follow-up time, smoking, and physical activity in all categories of prediabetes.

**Table 2:** **Association between gestational diabetes and the risk of prediabetic stages at the follow-up study**

	IIFG			IIGT			IFG + IGT			Prediabetes		
	(N = 267)			(N = 26)			(N = 40)			(N = 333)		
	HR	95% CI	***P***	HR	95% CI	***P***	HR	95% CI	***P***	HR	95% CI	***P***
Model 1	3.1	2.4-4.0	**<0.001**	7.8	2.9-21.6	**<0.001**	4.7	2.2-10.0	**<0.001**	4.0	3.1-5.1	**<0.001**
Model 2	3.2	2.5-4.3	**<0.001**	7.8	2.8-21.7	**<0.001**	3.7	1.7-7.9	**0.001**	3.8	3.0-4.9	**<0.001**
Model 3	3.0	2.3-4.0	**<0.001**	7.7	2.7-21.4	**<0.001**	3.5	1.6-7.6	**0.001**	3.6	2.8-4.6	**<0.001**
Model 4	3.1	2.4-4.1	**<0.001**	7.6	2.7-21.3	**<0.001**	3.1	1.4-6.9	**0.004**	3.7	2.8-4.7	**<0.001**

Cox proportional hazard model comparing the association of GDM with prediabetic stages vs NGT. IIFG, isolated impaired fasting glucose, IIGT, isolated impaired glucose tolerance, IFG + IGT, combined impaired fasting glucose and impaired glucose tolerance; HR, Hazard ratio: CI, confidence interval. P-values < 0.05 are indicated in bold font.

Model 1: unadjusted.

Model 2: Adjusted for age, BMI.

Model 3: Adjusted for age, BMI, parity.

Model 4: Adjusted with age, BMI, parity, smoking, physical activity.

The association between GDM at index pregnancy and subsequent hyperglycemia analyzed by linear regression analysis at the follow-up study is shown in Table 3. A strong association between GDM and fasting plasma glucose, 2hPG as well as glucose AUC was detected (*P* < 0.001 for all) which was attenuated after adjustment for age, BMI, follow-up time, parity, as well as smoking and physical activity. The association was further attenuated after adjustment for peripheral insulin sensitivity (Matsuda ISI) and attenuated or abolished after adjustment for insulin secretion (DI30).

**Table 3:** **Association between gestational diabetes and plasma glucose levels at the follow-up study**

Variable at follow-up study	B	SE	***P***	P*	P**	P***
Fasting plasma glucose (mmol/l)	0.27	0.03	**<0.001**	**<0.001**	**<0.001**	0.063
2-hour plasma glucose (mmol/l)	0.54	0.10	**<0.001**	**<0.001**	**0.011**	0.803
Glucose AUC (mmol/l)	83.04	10.84	**<0.001**	**<0.001**	**<0.001**	**0.011**

Linear regression analysis, individuals with diabetes diagnosed between index pregnancy and follow-up study are excluded, N = 385 controls and 474 patients with GDM. AUC, area under the curve. P-values < 0.05 are indicated in bold font.

*P,* unadjusted.

P*, adjusted for age, BMI, follow-up time, parity, smoking, physical activity.

P**, adjusted for age, BMI, follow-up time, parity, smoking, physical activity, Matsuda ISI.

P***, adjusted for age, BMI, follow-up time, parity, smoking, physical activity, Disposition Index.

Comparison of the women who proceeded to abnormal glucose tolerance (AGT, i.e. pre-diabetes or diabetes) after GDM in the index pregnancy to those who had GDM but maintained NGT during the follow-up, showed that the individuals with AGT were significantly older during the index pregnancy (*P* = 0.016) and at the time of the follow-up study (*P* < 0.001), but had also a longer follow-up time (*P* = 0.008) (Table 4). The women with AGT at follow-up had higher BMI in the first trimester of the index pregnancy and also at the follow-up study visit (*P* = 0.001 and *P* < 0.001, respectively). The mean difference in waist circumference was 7.0 cm between the progressors and non-progressors (94.0 cm and 87.0 cm, respectively, *P* < 0.001). Peripheral insulin sensitivity (Matsuda ISI) was significantly decreased in patients with AGT (*P* < 0.001), and insulin secretion (DI30) was markedly lower in the AGT group as compared to NGT group (*P* < 0.001).

**Table 4:** **Comparison of women with normal and abnormal glucose tolerance at the follow-up study visit among those affected by gestational diabetes**

	NGT (n = 228)		AGT (n = 246)		
	Mean	SD	Mean	SD	***P***
Age at delivery (years)	31.3	5.9	32.6	5.9	**0.016**
Number of deliveries	1.1	1.4	1.2	1.2	0.550
Age at follow-up study (years)	36.3	7.0	38.6	7.0	**<0.001**
Follow-up time (years)	5.2	4.0	6.3	4.5	**0.008**
Waist (cm)	87.0	12.3	94.0	12.6	**<0.001**
BMI in the first trimester (kg/m2)	25.6	4.6	26.9	4.6	**0.001**
BMI in the end of pregnancy (kg/m2)	30.1	4.4	31.2	4.5	**0.013**
Weight increment during pregnancy (kg)	12.3	6.2	12.2	5.9	0.993
BMI at follow-up study (kg/m2)	26.6	4.9	29.5	5.2	**<0.001**
Change in weight during follow-up time (kg)	1.6	7.3	5.5	8.1	**0.002**
Change in BMI during follow-up time (kg/m^2^)	1.1	2.7	2.6	3.0	**<0.001**
Current smoker (%)	21.8		15.6		0.090
Physical activity (%)	82.7		83.5		0.816
Family history of diabetes	79.9		81.5		0.669
HbA1c [mmol/mol (%)]	35 (5.3)	3	37 (5.5)	4	**<0.001**
Matsuda ISI (mg/dl, mU/l)	6.8	3.3	4.6	2.8	**<0.001**
Disposition Index	201.8	61.1	133.0	48.9	**<0.001**

NGT, normal glucose tolerance; AGT, abnormal glucose tolerance (includes both prediabetes and type 2 diabetes). Subjects diagnosed with type 2 diabetes between the index pregnancy and the follow-up visit were excluded. P-values < 0.05 are indicated in bold font.

## Discussion

Our long-term follow-up study shows that pre-diabetes is very frequent in women with a history of GDM. Gestational diabetes is strongly associated with both isolated impaired fasting glucose, isolated impaired glucose tolerance and also their combination. Patients with GDM are more insulin resistant than women who are normoglycemic during pregnancy. However, impaired insulin secretion due to beta cell failure seems to be the key defect leading to the deterioration of hyperglycemia after a pregnancy affected by GDM. Increasing waist circumference and weight during the postpartum follow-up period were significant risk factors for post-partum pre-diabetes or diabetes among women with a history of GDM, thereby suggesting that women with GDM could benefit from weight maintenance or weight loss interventions in the post-partum period.

Pre-diabetes is a preliminary stage of type 2 diabetes, albeit not all patients with pre-diabetes progress to overt disease. Approximately one in three of the individuals with pre-diabetes have been shown to progress to type 2 diabetes in previous studies [11]. Both IFG and IGT similarly associate with substantially increased risk of developing diabetes, and the risk is highest in the individuals with combined IFG and IGT. Apart from increased risk for diabetes, both IFG and IGT have been shown to associate with increased risk of coronary heart disease in women [15, 16]. In a previous Canadian study of 90 cases (with either GDM or impaired glucose tolerance during pregnancy) and 99 normoglycemic controls, GDM status was significantly associated with IFG at 15-years post-partum, but not with IGT or pooled pre-diabetes [17]. In our current study of 489 women with GDM and 385 normoglycemic controls, GDM was a significant predictor of all prediabetic stages separately (IIFG, IIGT, and IFG&IGT), and in combination at 7.3 years after the index pregnancy. In our study over 50% of the women with a history of GDM had developed either pre-diabetes or diabetes at follow-up (Figure 1), whereas the corresponding rate was half of that in the control group. Our findings are well aligned with previous studies (and meta-analyses/reviews) estimating the risk of diabetes following GDM.

Several lifestyle intervention trials have shown a remarkable reduction of type 2 diabetes risk in individuals with pre-diabetes. This can be achieved by dietary modifications and increased physical activity which ameliorate insulin resistance [12, 13]. Therefore, diagnosis of pre-diabetes at the postpartum follow-up after GDM pregnancy serves as a second alarm and recognizes a subgroup which should be in a focus of targeted prevention measures for type 2 diabetes.

In our follow-up study waist circumference was greater in women with a history of GDM when compared to controls, but interestingly so also in the women who proceeded to prediabetes or diabetes in the subgroup of women progressed with GDM to abnormal glucose tolerance versus those with GDM and normal glucose tolerance after pregnancy. Waist circumference is closely associated with lifestyle, and worryingly, some of the previous studies have shown that although most women would be aware of the risk of subsequent risk of diabetes after a GDM pregnancy, very limited lifestyle changes concerning the eating habits, weight reduction and/or the level of physical exercise unfortunately occur [18, 19]. Analysis of parous women in the Diabetes Prevention Program found that women with GDM had more difficulty complying with the intensive lifestyle intervention than parous women without GDM, and therefore women with GDM may require more help changing their dietary and physical activity behaviors for the purposes of diabetes prevention [20]. Taking all these facts into account, systematic follow-up and lifestyle interventions should be provided to women with a history of GDM pregnancy, and these should even be intensified if abnormal glucose tolerance is diagnosed at the follow-up post-pregnancy. Changing the course of the events before the development of overt disease helps to reduce the burden of disease associated with micro- and macrovascular complications, is economically cost-beneficial, and also indirectly benefits the offspring of GDM patients who are known to be in increased risk of overweight and metabolic syndrome [21].

Previous studies have shown that women with a history of GDM are on average more insulin resistant also in the non-pregnant state [22]. According to our results, insulin sensitivity was severely decreased in women with GDM also after pregnancy. Lifestyle factors including obesity, especially visceral obesity, and physical inactivity strongly associate with insulin resistance and type 2 diabetes [23]. During pregnancy, both maternal adiposity and the insulin-desensitizing effects of placental hormones lead to progressive insulin resistance which begins near mid-pregnancy and increases further during the third trimester. To maintain euglycemia, the pancreas compensates by secreting increased amounts of insulin. Thus, insulin resistance during pregnancy reveals limitations in insulin secretion and identifies a cohort of relatively young women with significant a defect in pancreatic beta cell function [24], and pregnancy has indeed hence been referred to as the metabolic window into the subsequent metabolic health in women. On the other hand, increasing insulin resistance and subsequent insulin hypersecretion eventually worsen the level of beta cell failure [9]. Our findings of the insulin secretion defect leading to the failure of compensatory mechanisms and development of hyperglycemia at both fasting and postprandial level are in agreement with this.

The subgroup analysis of the women with a history of GDM pregnancy showed that those who progressed to abnormal glucose tolerance (i.e. pre-diabetes or diabetes) were significantly older and had also a longer follow-up time (Table 4) which emphasizes the importance of long-term follow-up. Despite the guidelines of ADA [25] and the British National Institute for Clinical Excellence (NICE) [26] which recommend 1–3 yearly follow-up of glucose homeostasis post-partum, in practice the guidelines how to follow these women vary significantly and the uptake has remained low [27, 28]. Diabetes Prevention Programme study in US adults showing that women with GDM were less able to lose weight and to maintain exercise after the pregnancy [20]. Our results show that waist circumference as well as weight increment during the follow-up time were significant risk factors for developing pre-diabetes or diabetes after GDM pregnancy and also offer simple means to follow this risk group up by all healthcare providers. These factors are likely contribute to their increased risk of developing type 2 diabetes whilst also highlighting the importance of weight management in overweight and obese women with a history of GDM. The number of pregnancies affected by GDM relative to total number of pregnancies (all vs. only one of the pregnancies affected by GDM) was not significantly different between those who remained normoglycemic and those progressing to AGT in the current study (data not shown).

This study is not without limitations. The diagnostic criteria of GDM vary worldwide which makes it difficult to compare the findings across different populations. Furthermore, our study population was of relatively normal weight at the index pregnancy, included Caucasian women only and therefore the results cannot be generalized to other ethnic groups without caution [29]. The follow-up times of the study groups differed so that the follow-up time of controls was significantly longer compared to women with a GDM history. Thus, the incidence of prediabetes and type 2 diabetes is not an overestimate but rather on the contrary. Taking this into consideration, all regression analyses were adjusted with the follow-up time.

## Conclusions

In conclusion, in the current study we evaluated the long-term implications of GDM on glucose metabolism in a large cohort of Finnish women with GDM and healthy controls. GDM provides an important metabolic window to the subsequent glucose metabolism in women and of interest, we observed that pre-diabetic stages were alarmingly prevalent following GDM pregnancy. The timely recognition of pre-diabetes is of crucial importance for the prevention of type 2 diabetes before the development of overt disease. In the current study we observed that women with GDM history had decreased insulin sensitivity but hyperglycemia appeared after compensatory insulin secretion mechanism failed. Importantly, a subgroup analysis of women with GDM history showed that weight and development of central obesity were key risk factors of increased insulin resistance and subsequent progression to prediabetes and type 2 diabetes. This emphasizes the importance of lifestyle changes after gestational diabetes, especially avoidance of central obesity, which reduce insulin resistance and thereby stabilize or improve the beta cell defect.
